# Supplementary material for: Columnar processing of border ownership in primate visual cortex
Source: eLife. 2021 Nov 30;10:e72573. doi: 10.7554/eLife.72573 (PMC8631947; doi:10.7554/eLife.72573)
Supplement: Transparent reporting form [file elife-72573-transrepform1.pdf]

1st Floor
24 Hills Road
Cambridge CB2 1JP, UK

P 01223 855340
W elifesciences.org
T @elife

eLife's transparent reporting form

We encourage authors to provide detailed information *within their submission* to facilitate the interpretation and replication of experiments. Authors can upload supporting documentation to indicate the use of appropriate reporting guidelines for health-related research (see EQUATOR Network), life science research (see the BioSharing Information Resource), or the ARRIVE guidelines for reporting work involving animal research. Where applicable, authors should refer to any relevant reporting standards documents in this form.

If you have any questions, please consult our Journal Policies and/or contact us: editorial@elifesciences.org.

Sample-size estimation

- You should state whether an appropriate sample size was computed when the study was being designed
- You should state the statistical method of sample size computation and any required assumptions
- If no explicit power analysis was used, you should describe how you decided what sample (replicate) size (number) to use

Please outline where this information can be found within the submission (e.g., sections or figure legends), or explain why this information doesn't apply to your submission:

No sample-size calculations were performed. Sample sizes are standard in the field. For all analyses on population data, the relevant sample size (N units, N penetrations) is indicated in the figure legends or in the main text.

Replicates

- You should report how often each experiment was performed
- You should include a definition of biological versus technical replication
- The data obtained should be provided and sufficient information should be provided to indicate the number of independent biological and/or technical replicates
- If you encountered any outliers, you should describe how these were handled
- Criteria for exclusion/inclusion of data should be clearly stated
- High-throughput sequence data should be uploaded before submission, with a private link for reviewers provided (these are available from both GEO and ArrayExpress)

Please outline where this information can be found within the submission (e.g., sections or figure legends), or explain why this information doesn't apply to your submission:

eLife Sciences Publications, Ltd is a limited liability non-profit non-stock corporation incorporated in the State of Delaware, USA, with company number 5030732, and is registered in the UK with company number FC030576 and branch number BR015634 at the address 1st Floor, 24 Hills Road, Cambridge CB2 1JP | August 2014

In the manuscript a clear distinction is made between number of units/penetrations (biological replicates), different conditions (e.g. border ownership, contrast polarity, edge orientation) and the different trials that were obtained per unit per condition (technical replicates: typically 8-10, at least six were required for units to be included as candidate border ownership selective units (see Methods)). It is detailed in Methods how data from different trials were handled in the analyses (see definition of border ownership selectivity; border ownership reliability). We selected only one set of border ownership stimuli (orientation, position) for each unit in the analyses of time course, to avoid weighing units differently if a different number of stimulus sets with different edge orientations and positions were available. Inclusion/exclusion criteria for the evaluation of border ownership selectivity and for the laminar assignment are detailed in Methods.

Statistical reporting

- Statistical analysis methods should be described and justified
- Raw data should be presented in figures whenever informative to do so (typically when N per group is less than 10)
- For each experiment, you should identify the statistical tests used, exact values of N, definitions of center, methods of multiple test correction, and dispersion and precision measures (e.g., mean, median, SD, SEM, confidence intervals; and, for the major substantive results, a measure of effect size (e.g., Pearson's r, Cohen's d)
- Report exact p-values wherever possible alongside the summary statistics and 95% confidence intervals. These should be reported for all key questions and not only when the p-value is less than 0.05.

Please outline where this information can be found within the submission (e.g., sections or figure legends), or explain why this information doesn't apply to your submission:

Statistical analysis methods are specified in Methods for the different analyses. Summary statistics (center and dispersion/precision measures, 95% confidence intervals, exact values of N) are reported in main text and figure legends. Exact p-values are reported throughout the manuscript whenever possible, with identification of the statistical test used. Less common statistical tests (randomization tests; the bootstrap procedure that we adopted from Self et al. (2019)) are explained in detail in Methods.

(For large datasets, or papers with a very large number of statistical tests, you may upload a single table file with tests, Ns, etc., with reference to sections in the manuscript.)

Group allocation

- Indicate how samples were allocated into experimental groups (in the case of clinical studies, please specify allocation to treatment method); if randomization was used, please also state if restricted randomization was applied
- Indicate if masking was used during group allocation, data collection and/or data analysis

Please outline where this information can be found within the submission (e.g., sections or figure legends), or explain why this information doesn't apply to your submission:

The stimulus condition played in each trial was assigned pseudorandomly as explained in Methods.

Additional data files ("source data")

- We encourage you to upload relevant additional data files, such as numerical data that are represented as a graph in a figure, or as a summary table
- Where provided, these should be in the most useful format, and they can be uploaded as "Source data" files linked to a main figure or table
- Include model definition files including the full list of parameters used
- Include code used for data analysis (e.g., R, MatLab)
- Avoid stating that data files are "available upon request"

Please indicate the figures or tables for which source data files have been provided:

MATLAB figures with embedded data, and the data plotted as histograms in Figures 4D and 6D are publicly available on figshare (https://doi.org/10.6084/m9.figshare.16862299).
